# Chemical Exposures: Prostate Cancer and Early BPA Exposure

**Published:** 2006-09

**Authors:** Julian Josephson

In animal models, estrogens can drive carcinogenesis of the prostate and have long been suspected of playing a role in human prostate cancer. Scientists have hypothesized that prenatal exposure to estrogen-like compounds, including monomeric bisphenol A (BPA), may account for recent increases in rates of prostate cancer. Now a rat study by Gail Prins of the University of Illinois at Chicago Department of Urology, Shuk-Mei Ho of the University of Cincinnati Department of Environmental Health, and their colleagues provides the first evidence of a direct link between low-dose BPA exposure during development and later prostate cancer.

BPA initially was synthesized for use in also is used as a cross-linking compound in the manufacture of polycarbonate plastics and epoxy resins. It leaches from food and beverage containers and from dental sealants, although the latter currently are not thought to be a major source of exposure. Today, this hormonally active chemical is widespread in the environment, with detectable serum levels present in approximately 90% of humans in the United States and other industrialized countries, by Prins’s estimate. BPA concentrations measured in placental and fetal tissues may be fivefold higher than maternal serum levels, with higher levels found in male fetuses compared to females, according to Prins.

BPA has been in use for about 50 years in the industrialized world. Some investigators have proposed that widespread ingestion of monomeric BPA from polycarbonate food and drink containers might partially explain recent increases in prostate cancer rates. According to the American Cancer Society, rates have been on the rise since 1975. With the 1987 introduction of prostate-specific antigen testing, the newly enhanced ability to diagnose the disease caused incidence to spike to 240 age-adjusted cases per 100,000 men by 1992. After this “catch-up” period, rates dropped for three years, but are now back on the rise.

In the study, described in the 1 June 2006 issue of *Cancer Research,* groups of newborn rats were given high or low doses of estradiol or an environmentally relevant dose of BPA. The findings provide a molecular underpinning for potential long-term effects by showing changes in methyl groups on DNA that are responsible for turning genes on or off. For example, one key prostate gene that normally fuels cell growth during development stayed turned on in the prostates of male rats exposed to BPA or elevated estradiol from birth, says Prins. Such epigenetic alterations may leave a permanent impression on genes, possibly sensitizing the animal to developing diseases later in life.

One must exercise caution, however, in extrapolating the current rat findings to humans. How might one conduct an analogous program of research on men? The researchers consider such a program virtually impossible because 50 years or more typically would be required for results of early exposure to BPA to show up as prostate cancer.

Indeed, Rebecca Sokol, a professor of medicine at the University of Southern California, cautions against extrapolating human effects from rat studies. She does, however, note that unlike strong carcinogens that damage DNA profoundly, BPA appears to cause subtle changes that may pass from one generation to the next. She asks whether something is happening to alter genes seemingly without changing the genetic code itself.

Says Prins, “Our evidence shows that in an animal model, some genes are altered by the addition or removal of methyl groups on the DNA, which changes the ability of those genes to be transcribed and translated to proteins. It is possible that these effects may pass through generations as has been shown recently for sperm cells.” However, she adds, that analysis awaits future studies.

## Figures and Tables

**Figure:**
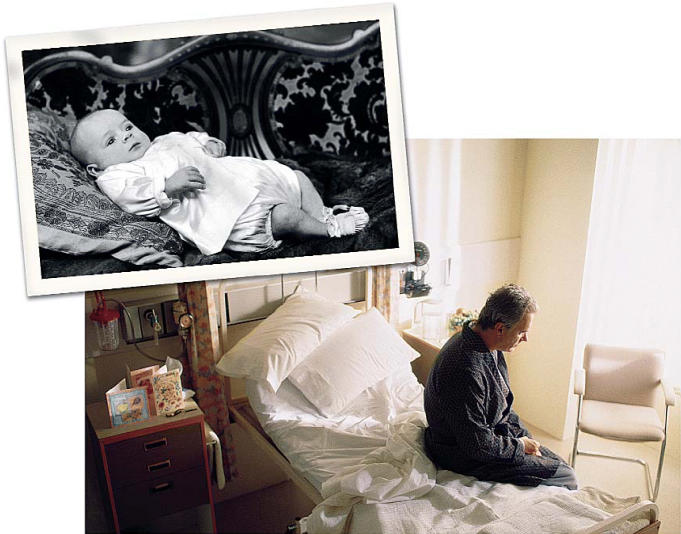
Disease through the ages New rat data link BPA exposure during critical periods of early development to later prostate cancer, raising compelling questions for research in humans.

